# Harnessing Residents’ Practice-based Inquiries to Enhance Research Literacy: The Thoughtful Reading of Evidence into Clinical Settings (T-RECS) Initiative

**DOI:** 10.5811/westjem.20921

**Published:** 2025-04-29

**Authors:** Emmagene Worley, Edward H. Suh, Liliya Abrukin, Michael DeFilippo, Jonathan J. Kamler, Mahesh Polavarapu, Peter C. Wyer

**Affiliations:** * Columbia University Irving Medical Center, Department of Emergency Medicine, New York, New York; † University of New Mexico, Department of Emergency Medicine, Albuquerque, New Mexico; ‡ Weill Cornell Medical Center, Department of Emergency Medicine, New York, New York

## Abstract

**Introduction:**

Research literacy is an important competency for all clinicians, but developing resident enthusiasm for it is difficult. At one academic emergency medicine (EM) residency program, we designed an innovative program to help residents improve literacy skills within a community of practice and use research literature to address clinical problems.

**Methods:**

A six-member faculty core team surveyed residents to assess their baseline experience with evidence-based medicine (EBM) and level of engagement with the medical literature. Interested residents joined an iterative curriculum development process that drew on previous EBM pedagogical experience and literacy theory. We developed a semi-structured approach that prioritizes using the reference frame of clinical applicability rather than research methodology. We held 90–120 minute sessions three times a year as part of the regular residency didactic conference; post-session evaluations with quantitative and qualitative elements were used to adjust subsequent didactics to refine the approach.

**Results:**

An average of 48 residents were in the EM training program during the nine sessions conducted during the study period. At baseline, residents had a high degree of exposure to EBM during medical school (94% of respondents) but low confidence in reading the medical literature (25%) or applying research to practice (10%). In contrast, they reported the novel program equipped them with skills to interpret literature and led to collective practice improvement. We found engagement was highest when residents led sessions based on inquiries that emerged out of their own training experience. Other positive factors included well-facilitated discussions between residents, relating questions to data-driven review of local practice patterns and addressing findings from free open access medical education (FOAMed) sources. The initial stages required significant team effort to design the pilot sessions, but later sessions were developed following the trajectory of resident inquiries using a minimally structured faculty consensus process and required less than 12 total faculty hours of commitment.

**Conclusion:**

An innovative program centered on residents’ practice-based queries of research literature appears to enhance learner enthusiasm for development of research literacy. Further development is needed to validate the overall effectiveness and generalizability of this approach.

## INTRODUCTION

Understanding the medical literature has always been an important skill for healthcare professionals, including physicians in clinical practice. Physicians are expected to stay abreast of emerging knowledge about new diseases, diagnostic approaches, and treatments to advance the care they provide to their patients. Facility with reading literature is crucial to properly applying it to local practice environments and individual patient needs.

The volume of medical literature has grown exponentially in recent years.^1^ Accordingly, entire ecosystems of resources have emerged that provide pre-appraisal and interpretation of research, including the collection of sources known as free open access medical education (FOAMed).^2^ Residents now need to know how to interpret and use both primary and secondary sources. However, the traditional methods of teaching research evaluation, such as critical appraisal checklists and statistical lectures, are of questionable benefit to clinically oriented learners and do not apply to FOAMed sources.^3^,^4^

We sought to develop a new approach to developing literacy, one that would foster a community of practice that engaged the medical literature in the context of resident experience. To distinguish this effort from traditional ones and emphasize our intended focus on application to practice, we named it “Thoughtful Reading of Evidence into Clinical Settings,” or “T-RECS.”

## METHODS

### Setting

This educational innovation was developed in an emergency medicine residency program affiliated with two urban, academic medical centers within one hospital system. This 48-trainee, four-year program during the study period was staffed with 12 residents per class, who covered emergency department (ED) sites treating more than 250,000 patient visits annually. Approval by the institutional review board was not sought as this was an educational initiative conducted entirely within the context of the standing didactic program, and individual subjects were not enrolled. Residents participated in the sessions as part of their weekly general didactic conference; the T-RECS organizing group did not influence which residents attended or took part.

### Initial Curricular Development

A core group of six faculty assembled to design and implement a new approach to teaching what has traditionally been called evidence-based medicine (EBM). The team included a residency assistant program director, departmental operations and quality improvement leaders, the director of clinical pathway development, and other faculty with experience in teaching EBM.

We developed a needs assessment survey (Supplement Material 1) to gauge baseline exposure to and facility with EBM, typical sources of medical literature, and related practice behavior. The survey was distributed electronically over the residency mailing list and completed anonymously by respondents via Qualtrics (Qualtrics International Inc, Provo, UT). Two residents were recruited into the curricular development team based on their responses to the survey. We considered various approaches to curriculum development, but there was clear consensus on making our focus the clinical application and contextualization of primary and secondary research reports. We also decided that maximizing learner engagement would take priority above an attempt to comprehensively cover concepts related to research design and statistical methodology.

### Pilot Session Planning

The resident participants shared clinical questions they had encountered during their practice and identified sources of information they had accessed to address those topics. Choosing a few topics that seemed promising from the perspective of engagement and available evidence, our group surveyed attending physicians on their personal practice in those subject areas to create linkage to local context. The core team then met to identify potential points of emphasis ranging from methodological and statistical to clinical applicability; we designed a 90-minute, Zoom-based pilot session incorporating the above that we hoped would stimulate rich, resident-led discussion. Residents were broken into small groups, each with a faculty facilitator. Group leaders led semi-structured discussion along the pre-specified points of emphasis, prioritizing dialogue rather than completion of the planned material. (Supplemental Material)

### Iteration and Refinement

After a positive response to the first session, T-RECS was scheduled three times a year as part of the regular weekly residency conference series. Subsequent sessions varied in length from 90–120 minutes. Initially, sessions were held virtually because of the COVID-19 pandemic but are now in person.

The approach was refined based on quantitative and qualitative feedback from post-session participant assessments and the observations of the leadership team. After each session, a short evaluation survey was distributed to the participants using a direct link to an anonymous survey. Results of these evaluations helped inform subsequent refinements to our protocol. Different variations were trialed, including covering different topics or papers in each small group, sharing results of faculty practice surveys, review of electronic health record data, and invitation of extra-departmental faculty to contextualize discussions. Enthusiasm for T-RECS has allowed continual recruitment of interested residents who can drive subsequent sessions.

## RESULTS

Nine T-RECS sessions have been held. Approximately half of the 48 residents were present at any given conference; over the course of the academic year all residents attend at least one session. In the baseline survey of trainees, nearly all (94%) respondents reported having education on EBM during medical school, with half of those recalling attending more than six lectures on the subject. Yet only 25% of respondents felt “very comfortable” interpreting research studies, and only 10% were confident in their ability to judge the applicability of a study to their own clinical practice.

On the other hand, post-session evaluations conducted after conference have revealed consistent positive sentiment toward T-RECS. Compiled results from post-session evaluations after the first five sessions are reported below in the Table. While subsequent sessions did not evaluate the same questions, there has been continued high satisfaction the overall approach and level of emphasis on statistical concepts. Qualitative feedback from residents have revealed clear themes. Clinical relevance is seen as a major positive, as are small-group discussions, effective faculty facilitation, a pivot away from statistics, and the use of resident-discovered resources. The feedback received from the pilot session are reported in full in the Supplemental Materials as a sample of the comments received. Feedback from faculty leadership reported similar findings, with emphasis on the observation that engagement was best when topic selection was driven by spontaneous resident inquiries into issues and decisions that they had found frustrating in clinical practice.

### Current Framework of T-RECS Sessions

While emphasis on formal critical appraisal tools is of little interest to our residents, the need for some basic shared framework for group reading and discussion became clear. The PICO formulation (patients, intervention, comparison, outcomes) emerged as an ideal structure for this purpose. Originally developed as a tool to guide systematic evidence reviews, PICO is usually used as a framework for formulating clinical research questions.^6^, ^7^ We use the acronym as a guide to identifying the fundamental points of comparison between reported research results and clinical practice. Active identification of the PICO components while reading creates a scaffold by which the applicability to practice can be integrated into each reader’s own medical knowledge and context.

### Present Approach to T-RECS Session Planning

Preparation time for each conference has significantly shortened with experience and now averages less than 12 faculty hours per session between 2–3 faculty members. This translates to an approximate faculty salary cost of $2,400 per session, or $7,200 per academic year to maintain the program. Session planning begins by soliciting clinical topics from residents based on actual searches they have recently independently conducted and the sources they had used. Faculty identify the potential teaching points available in the primary research reports, secondary appraisals, and the search process. These considerations are condensed into a set of prompts and discussion points that are used to facilitate small-group conversation.

## DISCUSSION

Nurturing emergency physicians to be adept at informing practice through research enhances patient care. Such clinicians are better equipped to navigate the dynamic landscape of medicine. But teaching research literacy is difficult. Prior attempts at our institution to address this using various educational formats were less successful.^8^ They relied on traditional EBM methods that skewed heavily toward methodology and statistics and reflected the “educational prescription” model that had been promoted in early EBM literature.^7^ In those models, residents are required to formulate research questions and conduct quasi-systematic reviews. Residents find such exercises artificial and disconnected from their clinical education.^9^

The T-RECS approach instead seeks to enhance “literacy.” Rather than try to teach biostatistical methods to aspiring clinicians, T-RECS draws on the activities and realities in which the learners are actually immersed. It fosters the ability to integrate information from the research literature with knowledge from personal and shared practice experience. It constitutes a program for resident empowerment akin to the literacy methods developed by the Brazilian educator Paulo Freire, using the advantages conferred by team-based, small-group learning.^10^, ^11^

The T-RECS approach coheres with the Accreditation Council for Graduate Medical Education practice-based learning and improvement (PBLI) competency, particularly as the EM milestones call for literature review in relationship to practice issues encountered by residents.^12^ The PBLI competency conceives such review as a core aspect of reflective practice.^13^, ^14^ On the other hand, T-RECS is distinct from journal club. In most programs, journal club is held outside clinical or educational hours, which emphasizes its social function.^15^ It can serve as a means of keeping up with the literature and acquisition of critical appraisal skills, but it is typically expert-led and responds to what is important to publishers of medical journals. 16 In contrast, T-RECS is resident-driven and focuses on inquiries arising from their actual practice experience. Although a curricular approach has been advocated for journal clubs,^17^ we avoided imposing any such framework in T-RECS to maximize the immediacy of resident-driven topics and literature.

Therefore, we worked with organically emerging topics, while being mindful to expose learners to a breadth of concepts and skills. Further details on sessions are available in the online Supplement. The FOAMed resources are often cited and heavily influence their perspectives, and our survey confirmed that they are used more frequently than primary sources in connection with practice-based inquiries. We drew on them to frame the T-RECS session alongside primary sources they are based on.

## LIMITATIONS

There are some limitations to this approach and our findings thus far. Substantial faculty time and effort were required to initiate the program, although this has diminished significantly over time. We benefited from an established emphasis on facilitated small-group learning within the program, which may not be available in all settings. The T-RECS approach also relies on meaningful resident engagement; while participation has been robust, maintenance of a community of practice, a culture of shared inquiry and practice transformation, is critical for the sustainability of the program. Finally, T-RECS is aimed at the clinically oriented trainee. Although most residents in our program fit this profile, systems with a higher percentage of research-oriented learners may desire more emphasis on methodology and statistics.

## CONCLUSION

A resident-driven approach to enhancement of literature reading skills that emphasizes applicability to practice and context over statistics and research methodology has resulted in a positive learner response. Further development and exploration of its applicability to other EM programs appear warranted.

## Figures and Tables

**Table:** Results of voluntary post-evaluation survey results. Responses were collected as Likert-type items, from 1 (most negative) to 5 (most positive) and are reported as mean and standard deviation, except for “emphasis on statistical concepts,” which was collected as a 3-choice response: “too little”; “too much”; or “just right (reported as percentage response.)

	N	“The session increased my ability to evaluate the relevance of research to clinical practice”	“Discussion of collective clinical practice enhanced the session”	“The facilitation was effective”	“The emphasis on statistical concepts was ‘just right’”	“The *additional element improved the session”	*Additional element
Session 1	13	3.9 (1.1)	3.9 (1.1)	4.2 (0.9)	46.2%	4.4 (0.8)	FOAMed piece
Session 2	13	3.8 (0.8)	3.8 (1.2)	4.2 (0.9)	92.3%	3.9 (1.0)	FOAMed piece
Session 3	13	3.3 (1.2)	3.9 (1.0)	3.7 (1.4)	53.8%	3.2 (1.1)	Outside content expert
Session 4	13	4.1 (1.0)	4.5 (0.9)	4.5 (0.8)	76.9%	4.1 (1.1)	Survey of faculty opinion
Session 5	29	4.5 (0.7)	4.7 (0.5)	4.8 (0.4)	96.6%	4.5 (0.6)	Faculty vs faculty debate

*FOAMed*, free open access medical education
